# Sex chromosome loss and the pseudoautosomal region genes in hematological malignancies

**DOI:** 10.18632/oncotarget.12050

**Published:** 2016-09-15

**Authors:** Stephanie Weng, Samuel A. Stoner, Dong-Er Zhang

**Affiliations:** ^1^ Moores Cancer Center, University of California San Diego, La Jolla, CA, USA; ^2^ Department of Pathology and Division of Biological Sciences, University of California San Diego, La Jolla, CA, USA

**Keywords:** sex chromosome, hematological malignancy, pseudoautosomal region, sex chromosome loss, haploinsufficiency

## Abstract

Cytogenetic aberrations, such as chromosomal translocations, aneuploidy, and amplifications, are frequently detected in hematological malignancies. For many of the common autosomal aberrations, the mechanisms underlying their roles in cancer development have been well-characterized. On the contrary, although loss of a sex chromosome is observed in a broad range of hematological malignancies, how it cooperates in disease development is less understood. Nevertheless, it has been postulated that tumor suppressor genes reside on the sex chromosomes. Although the X and Y sex chromosomes are highly divergent, the pseudoautosomal regions are homologous between both chromosomes. Here, we review what is currently known about the pseudoautosomal region genes in the hematological system. Additionally, we discuss implications for haploinsufficiency of critical pseudoautosomal region sex chromosome genes, driven by sex chromosome loss, in promoting hematological malignancies. Because mechanistic studies on disease development rely heavily on murine models, we also discuss the challenges and caveats of existing models, and propose alternatives for examining the involvement of pseudoautosomal region genes and loss of a sex chromosome *in vivo*. With the widespread detection of loss of a sex chromosome in different hematological malignances, the elucidation of the role of pseudoautosomal region genes in the development and progression of these diseases would be invaluable to the field.

## INTRODUCTION

Chromosomal abnormalities are frequently detected in cancers. These abnormalities include events such as aneuploidy, chromosomal duplications, deletions, inversions, and translocations. Genomic regions containing critical proto-oncogenes and tumor suppressors are often targeted by these events. The subsequent alteration of the copy number, expression, or sequence of these proto-oncogenes and tumor suppressors ultimately contributes to or promotes tumorigenesis. Numerous studies have largely expanded our understanding of the mechanisms underlying various chromosomal abnormalities and their impact on cancer development and progression [1]. However, there remain chromosomal aberrations that have not been investigated as thoroughly, such as sex chromosome abnormalities.

Sex chromosome aberrations have been reported in a variety of malignancies. One of the most commonly observed sex chromosome aberrations is aneuploidy or loss, which has been reported in a variety of cancers, including lung [2], pancreatic [3], breast [4, 5], colorectal [6], and hematological cancers [7]. These observations suggest that tumor suppressor genes may reside on the sex chromosomes, and their loss may contribute to cancer development or progression.

## LOSS OF A SEX CHROMOSOME (LOS) IN HEMATOLOGICAL MALIGNANCIES

Loss of a sex chromosome (LOS) is frequently observed in hematological malignances. In a study of 868 patients with hematologic diseases, 5.1% of patients exhibited LOS [8]. Acute myeloid leukemia (AML) patients displayed the highest frequency of LOS, 9.5%, compared to a range of 0-6% for acute lymphoid leukemia (ALL), chronic lymphoid leukemia (CLL), or chronic myeloid leukemia (CML) patients [8]. Loss of the Y chromosome has also been observed in myelodysplastic syndrome (MDS) [9].

Cytogenetic studies of normal non-malignant bone marrow and peripheral blood cells indicate that loss of a sex chromosome or sex chromosome loss (SCL) is a phenomenon associated with aging [10–12]. Although aneuploidy generally increases with age [13, 14], LOS has been reported to occur at a rate 10-fold greater than autosomal chromosome loss [15]. Additionally, LOS is observed at higher frequencies and at younger ages in cancers. Several studies of childhood ALL have reported LOS in patient leukemia cells [16, 17]. Another larger cytogenetic study of 270 AML patient samples revealed that LOS was observed in 22 patients. Of these 22 patients, 50% were under the age of 14, 40% were aged 14 to 50, and 10% were aged above 50 [18], which further corroborates that LOS occurs at younger ages in cancers. Additionally, in a search for cytogenetic aberrations that may confer adverse risk in childhood ALL, LOS was correlated with increased risk [17]. Altogether, these observations find a positive correlation between LOS and hematological malignancies, implicating LOS as a risk factor for cancer development and prognosis.

Although LOS is common in hematological malignancies, only 1.8% of patients exhibit LOS as a sole genetic abnormality [8], and LOS is frequently observed with additional mutations [7]. However, LOS is typically observed in clonal populations, which further suggests that this cytogenetic aberration is favored for oncogenesis [19]. These findings, along with the fact that hematological malignancies are rarely associated with Turner syndrome, indicate that LOS may not be sufficient for malignant transformation, but is likely to serve as a cooperating event [7, 20]. In support of this is the frequent co-occurrence of LOS in around 32-59% of t(8;21) AML patients, which is much higher compared to other types of AMLs and leukemias [21, 22]. Unlike other oncogenic translocations, t(8;21) is insufficient for leukemogenesis and requires additional cooperating mutations, which LOS may serve as [23].

Altogether, these findings support that the sex chromosomes may harbor critical tumor suppressor genes, which when lost upon LOS, may contribute to disease development. However, the mechanistic contributions of LOS to disease development are not currently understood and have yet to be thoroughly investigated.

## PSEUDOAUTOSOMAL REGIONS (PARS)

The X chromosome is composed of just over 156 million base pairs, which contain 1,805 genes. The Y chromosome is roughly a third of the size and is composed of around 57 million base pairs, which contain 458 genes. Females possess both a maternal and paternal X chromosome, whereas males possess a maternal X chromosome and a paternal Y chromosome [24]. In females, one of the X chromosomes is subjected to high levels of DNA methylation to generate repressive heterochromatin, resulting in X-inactivation and gene silencing [24].

**Figure 1:**
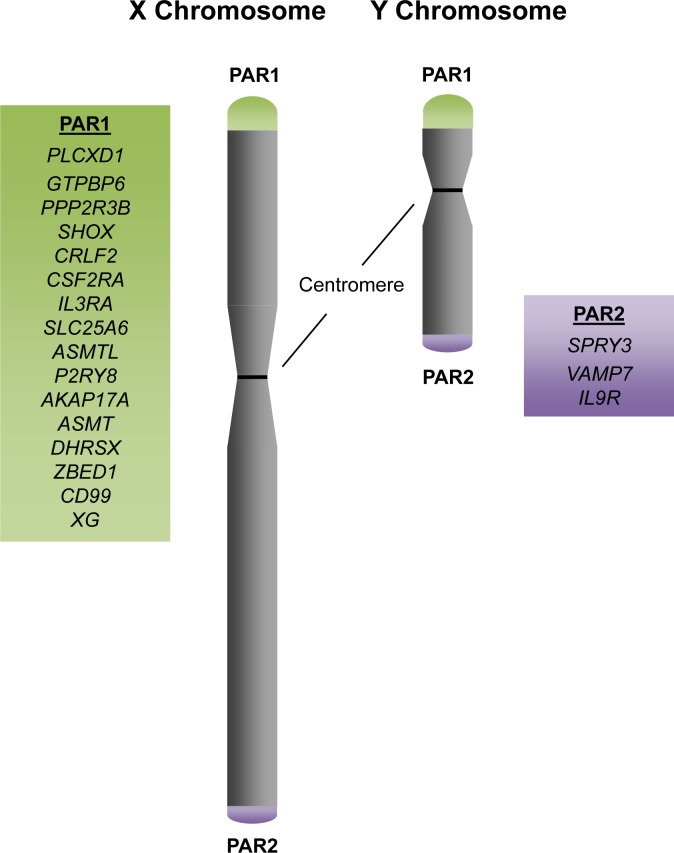
The Pseudoautosomal Regions and Genes Schematic of the X and Y sex chromosomes with the distal pseudoautosomal regions (PARs). PAR1 contains 16 genes, with *PLCXD1* as the furthermost PAR1 gene at the distal telomeric end and *XG* at the boundary of PAR1 at the centromeric end. PAR2 contains 3 genes, with *SPRY3* at the centromeric boundary and *IL9R* at the distal telomeric end.

Although the X and Y sex chromosomes are highly divergent in sequence, the pseudoautosomal regions (PARs) are homologous between the two chromosomes, escape X-inactivation, and facilitate proper pairing and segregation of the sex chromosomes during meiosis [25]. There are two main pseudoautosomal regions (PAR1 and PAR2) on the sex chromosomes, and they are localized to the distal ends of the p and q chromosomal arms, respectively [26] (Figure 1). PAR1 is 2.6Mb and contains 16 genes, whereas PAR2 is 360Kb and contains 3 genes and 1 pseudogene, *CXYorf1* or *WASH6P*. In 2013, a third pseudoautosomal region (PAR3) was identified and reported to have arisen from duplication and transposition of the q21.3 region of the X chromosome, which contains the genes PCDH11X/Y and TGIF2LX/Y, to the p11.2 region of the Y chromosome [27]. Because PAR3 is only observed in approximately 2% of the general population, we focus on PAR1 and 2 in this review.

Because the PARs escape X-inactivation, PAR genes are biallelically expressed in both females and in males. In the case of LOS, PAR genes suffer from haploinsufficiency and are the only genes commonly affected in both males and females with LOS. Consequently, the PAR genes are the most likely candidates as common tumor suppressors amongst the X and Y chromosomes.

Interestingly, PAR genes are not commonly mutated in most hematological malignancies [29]. The exception is diffuse large B-cell lymphomas, in which amplifications, deletions, and missense mutations of individual PAR genes have been identified in 2-10% of the 48 patient samples sequenced [29]. In the remaining hematological malignancies, perturbations to PAR genes are found in less than 1% of patient samples [29]. However, LOS, which results in haploinsufficiency of PAR genes, may contribute to disease development by reducing gene dosage of potential tumor suppressor genes. Further substantiating that PAR genes may possess tumor suppressive functions is the observation that pseudoautosomal regions are frequent targets for loss of heterozygosity (LOH) in cancers. LOH in a region of the PAR, which results in deletion of *SHOX, CRLF2, and CSF2RA*, has been observed in mantle cell lymphoma [28].

While LOS is frequently observed in hematological malignancies, especially AMLs, there is a shortage of studies examining PAR genes in hematological disease development and progression. Moreover, many of the PAR genes are not well-characterized. Further mechanistic studies of the role of PAR genes in normal and malignant hematological conditions will contribute immensely to our understanding of how LOS may be involved in oncogenesis. Additionally, systematic knockout or enforced expression of individual or a combination of PAR genes in the hematopoietic system will greatly elucidate the function of these genes in hematological diseases.

Here, we review what is currently known about the PAR genes in the hematopoietic system and discuss the implications for LOS and haploinsufficiency of PAR genes in the development of hematological malignancies.

## PAR GENES IN THE HEMATOPOIETIC SYSTEM

The nineteen PAR genes have a broad range of diverse functions. Some of these genes act as tumor suppressors or proto-oncogenes. Others have been reported to function as both, depending on cellular context, which highlights the need for examining the roles of PAR genes in individual hematological conditions (Table 1).

**Table 1:** Overview of PAR genes and their functions

Category	Gene Name	Gene function	References
Tumor suppressors	*PPP2R3B*	Subunit of PP2A. PP2A is inhibited by BCR-ABL1 and FTY720 has been reported to activate PP2A.	[30–34]
	*SLC25A6*	Component of the mitochondrial permeability transition pore (MPTP), which transports ADP in and ATP out of the mitochondrial matrix, to regulate metabolism and apoptosis. Downregulated in HL-60 and K562 leukemia cell lines.	[35–37]
	*SHOX*	Mutations and deletions observed in Hodgkin's lymphoma and mantle cell lymphoma.	[28, 38–40]
	*ASMT*	Acetylseratonin O-methyltransferase, catalyzes final step of melatonin synthesis. Melatonin induces cytotoxicity in chemoresistant leukemia cell lines.	[41–45]
	*DHRSX*	Oxidoreductase enzyme that regulates autophagy.	[46, 47]
Proto-oncogenes	*CRLF2*	Receptor for thymic stromal lymphopoietin (TSLP), which activates JAK-STAT signaling. Overexpression is observed in ALL.	[48–52]
	*IL3RA*	Receptor for IL-3, which stimulates proliferation of myeloid cells. Upregulated in AML, MDS, Hodgkin's lymphoma, and other hematological malignancies.	[53–58]
Context-Dependent	*CSF2RA*	Receptor for GM-CSF, which stimulates proliferation, survival, and differentiation. Upregulation is observed in CMML and JMML. Loss of GM-CSF signaling has also been reported to promote t(8;21) leukemogenesis.	[59–64]
	*P2RY8*	Purinergic orphan receptor of the G-protein coupled receptor family. *P2RY8* upregulation has been reported in leukemias. Loss of function mutations have been reported in lymphomas.	[44, 65, 66]
	*IL9R*	Receptor for IL9, which stimulates proliferation and inhibits apoptosis. Upregulation of IL9R has been observed in leukemias and lymphomas. IL9 has also been reported to possess tumor suppressive functions via the activation of immunosurveillance.	[67–69]
	*CD99*	Cell surface glycoprotein expressed on hematopoietic cells. CD99 regulates T-cell adhesion and apoptosis. Anti-CD99 antibodies have also been reported to promote apoptosis.	[70–73]
Inactive	*SPRY3*	One of the four mammalian Sprouty proteins.	[74–76]
	*VAMP7*	Vesicle associated membrane protein found in membranes of late endosomes and lysosomes. Aids in fusion of vesicles to target membranes, which is also required for cytotoxic granule release in NK cells.	[74, 75, 77–80]
Uncharacterized	*PLCXD1*	*PLCXD1* was found to be methylated in melanoma cell lines.	[81]
	*ASMTL*	Point mutations in *ASMTL* have been reported in childhood ALL.	[82, 83]
	*AKAP17A*	Binds to type I and II PKA and interacts with splicing factors to regulate alternative splicing.	[84, 85]
	*ZBED*	Transcription factor that regulates DNA replication and cell proliferation genes.	[86, 87]
	*XG*	Blood group antigen, shares 48% sequence homology with CD99.	[88–91]
	*GTPBP6*	Putative GTP-binding protein, contains 4 GTP-binding sites.	[92, 93]

### Tumor suppressors

#### PPP2R3B

**Figure 2:**
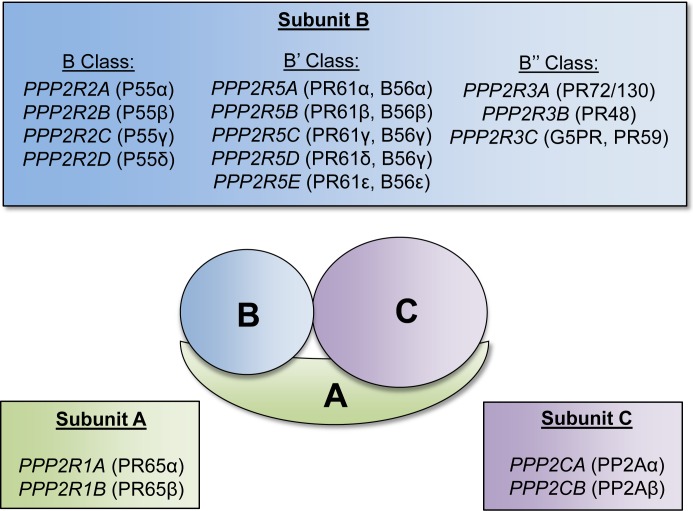
Protein phosphatase 2A subunits and respective encoding genes Schematic of the serine-threonine protein phosphatase (PP2A) complex. The PP2A complex is composed of three subunits: A, B, and C. Each respective subunit is encoded by the listed genes. The A and C subunits form a catalytic core, and the B subunit is involved in determining substrate specificity.

There are several PAR genes that have been implicated in possessing tumor suppressive functions. One of these genes is *PPP2R3B* (*NYREN8, PPP2R3L, PPP2R3LY, PR48*) which encodes one of the subunits of protein phosphatase 2A (PP2A). PP2A is a holoenzyme composed of A, B, and C subunits, each of which is encoded by several genes [30] (Figure 2). The A and C subunits form a catalytic core, and the B subunit is involved in determining substrate specificity. PP2A functions as a serine-threonine phosphatase and regulates various intracellular signaling pathways, and is generally regarded as a tumor suppressor [30]. Deletion, mutation, or inactivation of PP2A is frequently observed in various cancer types [30]. The oncofusion protein BCR-ABL1, which is generated from t(9;22), has been reported to inhibit PP2A activity [31]. Interestingly, inhibition of PP2A was found to be required for proliferative and pro-survival signals in CML [31]. Furthermore, the sphingosine analog FTY720, which has been demonstrated to activate PP2A, was found to reduce cell proliferation and induce apoptosis in models of CML, as well as on the Kasumi-1 t(8;21) AML cell line [32, 33]. Because PP2A is composed of multiple subunits encoded by 17 different genes, there are multiple avenues for altering the expression or activity of this phosphatase. Analysis of the genes encoding the different subunits of PP2A revealed that one of the genes encoding the A subunit, *PPP2R1B*, is downregulated in AML cells, and two genes encoding the B subunits, *PPP2R3B* and *PPP2R2C* are not expressed in AML cells [34]. Altogether, reduction in *PPP2R3B* gene dosage via LOS may result in reduced overall activity of PP2A, which could promote increased proliferation and confer survival advantage for leukemic cells.

#### SLC25A6

In a screen for genes required for TNF-induced apoptosis in the MCF-7 breast cancer cell line, Solute carrier family 25 member 6 (*SLC25A6, AAC3, ANT3*) was identified [35]. In this study, SLC25A6 was found to be necessary for apoptosis induced by TNF and oxidative stress, but not other types of apoptosis. SLC25A6 is a member of a family of genes that encode a component of the mitochondrial permeability transition pore (MPTP), which is critical in regulating cellular energy metabolism and apoptosis [35]. It functions as a transporter of ADP from the cytosol into the mitochondrial matrix and ATP from the mitochondrial matrix to the cytosol. Exogenous expression of SLC25A6 in HeLa cells increased sensitivity to ATRA-induced apoptosis [36]. SLC25A6 has also been reported to facilitate apoptosis induced by camptothecin, a cytotoxic agent, and knockdown of *SLC25A6* impairs apoptosis. Interestingly, *SLC25A6* was downregulated in several cancer cell lines upon treatment with camptothecin, including the leukemia cell lines HL-60 and K562, suggesting that reduced levels of *SLC25A6* may aid in conferring resistance to camptothecin-induced apoptosis [37]. Although the functions of SLC25A6 in hematopoiesis or leukemogenesis are not well characterized, these data indicate that it may serve as a tumor suppressor through its role in activating apoptosis.

#### SHOX

Short stature homeobox (*SHOX, GCFX, PHOGY, SS*) regulates bone growth and maturation. Several groups have reported gender concordance in siblings with Hodgkin's lymphoma [38, 39]. Additionally, Hodgkin's lymphoma has been found to occur in association with Leri-Weill dyschondrosteosis, a bone growth disorder that results in shortened arm and leg bones, which is caused by deletions or mutations of the PAR gene *SHOX*. These findings led to the hypothesis that disruption to *SHOX* may be linked to the development of Hodgkin's lymphoma [40]. Additionally, deletion of *SHOX* has also been reported in mantle cell lymphoma [28].

#### ASMT

**Figure 3:**
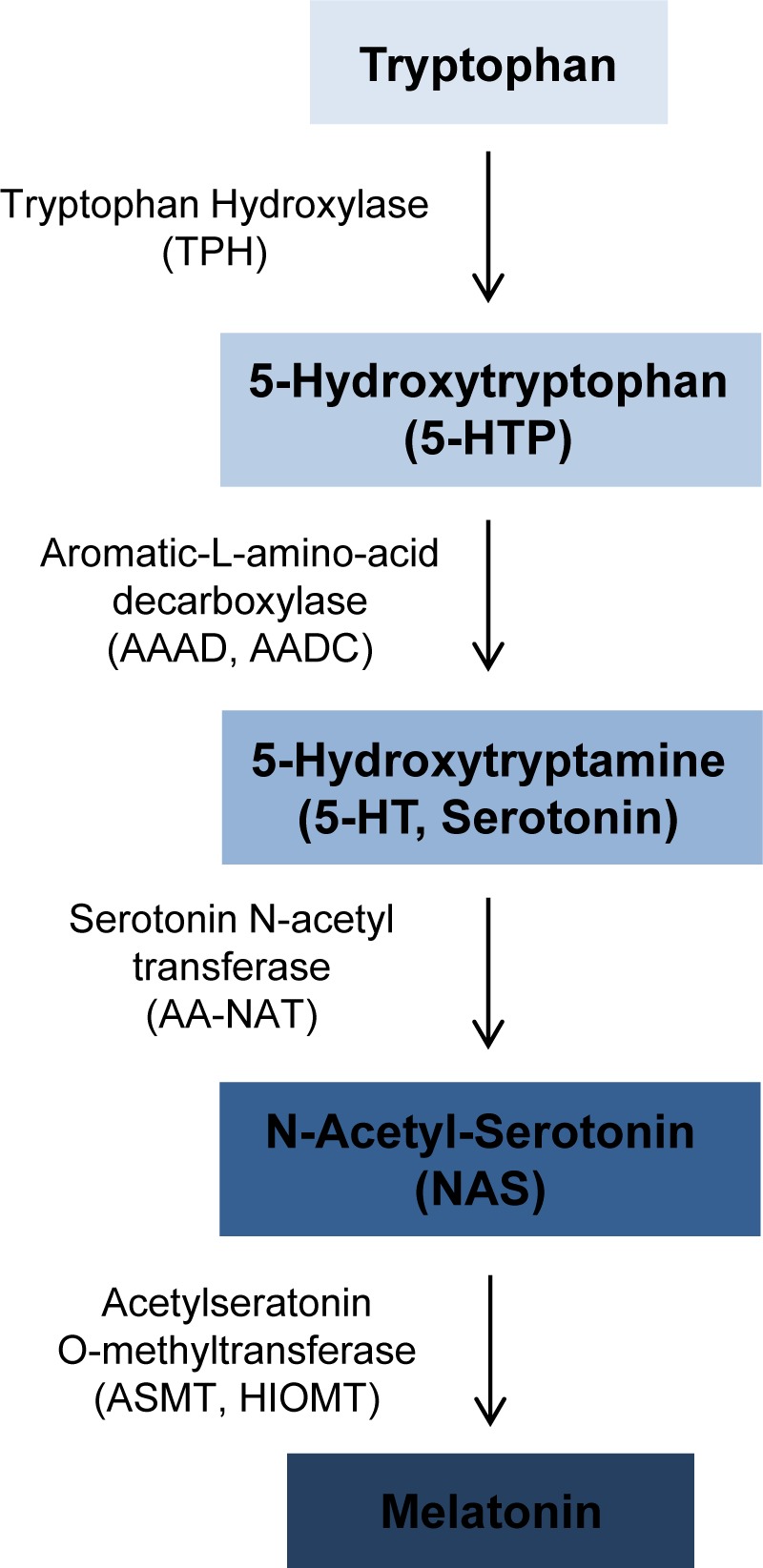
Overview of melatonin synthesis Melatonin is synthesized from tryptophan. The enzymes catalyzing each synthesis step are shown to the left of the arrows. The PAR1 gene *ASMT* catalyzes the final step in melatonin synthesis to convert N-Acetyl-Serotonin to melatonin.

Melatonin is an antioxidant that easily passes through cell membranes. Treatment of several human leukemia cell lines, including CMK, Jurkat, MOLT-3 and MOLT-4, with melatonin resulted in cytotoxic effects [41, 42]. *ASMT* (*ASMTY*, *HIOMT*, *HIOMT*) encodes acetylseratonin O-methyltransferase, an enzyme that catalyzes the final step in melatonin synthesis (Figure 3). Melatonin is predominantly synthesized in the pineal gland; however, melatonin has also been found to be synthesized by human bone marrow cells and several human hematopoietic cell lines [43]. *ASMT* expression is highest in T lymphocytes [44], and ASMT enzyme activity was found to be decrease in B lymphoblastoid cell lines derived from patients with ASMT mutations. Additionally, a study of clofarabine-resistant lymphoblastic leukemia cell lines revealed that melatonin treatment induced cytotoxic effects, and resulted in increased total H3 and H4 acetylation in these cells [45]. These findings indicate that melatonin can induce cytotoxicity of leukemia cells, and loss of function mutations or haploinsufficiency of *ASMT* may reduce melatonin levels and enhance cell survival.

#### DHRSX

In normal cells, autophagy is a critical regulator of cellular homeostasis. Autophagy is a lysosome-mediated mechanism to degrade intracellular components and it can act as a tumor-suppressive mechanism, but also a chemo-resistance mechanism [46]. It has been reported that during early stages of cellular transformation, autophagy is disrupted or suppressed, which results in increased genomic instability. Dehydrogenase/reductase (SDR family) X-linked (*DHRSX, DHRSY, DHRSXY, DHRS5X, DHRS5Y, SDR7C6, CXORF11, SDR46C1*) is a member of the oxidoreductase enzyme family. Ectopic expression of DHRSX increased starvation-induced autophagy, and knockdown of DHRSX reduced autophagy in HeLa and U2OS cells [47]. Therefore, deletion or loss of *DHRSX* due to LOS could reduce autophagy and leave cells vulnerable to genomic insults that promote cellular transformation.

### Proto-oncogenes

Some PAR genes have also been reported to possess proto-oncogenic properties in the hematopoietic system. Interestingly, these genes encode cytokine receptors.

#### CRLF2

**Figure 4:**
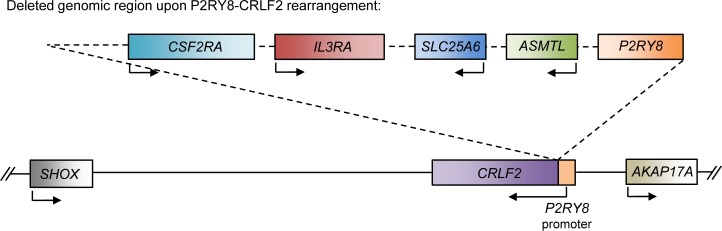
Schematic of the P2RY8-CRLF2 rearrangement Schematic of the affected genomic region upon the *P2RY8-CRLF2* rearrangement. This genomic deletion results in the fusion of the *P2RY8* promoter to the *CRLF2* gene, which results in upregulation of *CRLF2*. Additionally, the coding region of *P2RY8* is deleted, along with *ASMTL*, *SLC25A6*, *IL3RA*, and *CSF2RA*.

*CRLF2* (*TSLPR*) encodes the cytokine receptor-like factor 2, a type I cytokine receptor. It forms a heterodimer with IL7R (Interleukin 7 Receptor) to bind to its ligand, thymic stromal lymphopoietin (TSLP), and activates JAK-STAT signaling [48]. Deletions in the PAR1 region have been detected in various types of ALL and results in the fusion of the noncoding region of exon 1 of *P2RY8* to the *CRLF2* gene, which causes *CRLF2* expression to be driven by the *P2RY8* promoter [49–51] (Figure 4). This fusion has been reported to increase *CRLF2* expression by up to 10-fold, and results in increased activation of downstream signaling pathways, such as the JAK-STAT pathway, that promote proliferation of B cell precursors [52]. B-ALL patients with JAK2 mutations also frequently overexpress *CRLF2*, which leads to cytokine-independent growth [49]. Additionally, patients with high expression of *CRLF2* have poor prognoses. However, the deletion leading to the fusion of *P2RY8* and *CRLF2* also results in the deletion of several genes (*P2RY8*, *CSF2RA*, *IL3RA*, *SLC25A6*, and *ASMTL*) whose potential roles as tumor suppressors should not be discounted.

#### IL3RA

The other proto-oncogenic receptor is the Interleukin 3 receptor alpha (*IL3RA* or CD123), which is highly expressed on monocytes, macrophages, alveolar macrophages, and dendritic cells [44]. The IL-3 receptor is composed of the CD123 alpha receptor and the CD131 common GM-CSF beta receptor. Although IL-3 signaling promotes differentiation of hematopoietic stem/progenitor cell, it also stimulates proliferation of myeloid cells and is often used as a growth factor for myeloid cell lines [53]. CD123 has been found to be highly expressed in a variety of hematological malignancies, including AML, myelodysplastic syndrome, Hodgkin's lymphoma, hairy cell leukemia, systemic mastocytosis, plasmacytoid dendritic cell (pDC) leukemia, chronic myeloid leukemia, and B-cell acute lymphoblastic leukemia [54–56]. Therefore, CD123 has become an attractive hematopoietic cancer cell biomarker to target. Anti-CD123 therapies, such as monoclonal antibodies and chimeric antigen receptor (CAR) T-cells, have shown promise in mouse models, and are now being investigated clinically for therapeutic efficacy in various hematological malignancies [56–58].

### Context-dependent PAR genes

Several of the PAR genes cannot be as readily classified as tumor suppressors or proto-oncogenes, due to conflicting discoveries in varying cellular contexts.

#### CSF2RA

**Figure 5:**
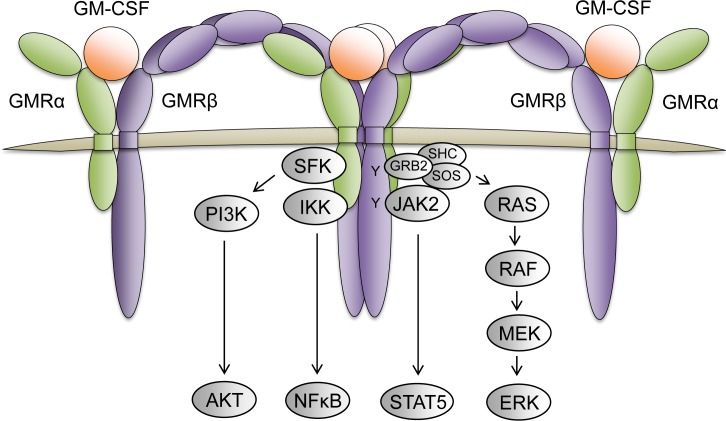
The GM-CSF receptor complex and its downstream signaling pathways The GM-CSF receptor is a dodecamer complex composed of 4 GMRα receptor subunits (green), 4 GMRβ receptor subunits (purple), and 4 GM-CSF molecules (orange). Upon GM-CSF ligand binding and receptor oligomerization, the juxtaposed GMRβ subunits bound to JAK2 promotes receptor transphosphorylation to initiate activation of various signaling pathways (grey) that regulate cellular proliferation, survival, and differentiation.

Colony stimulating factor 2 receptor alpha (*CSF2RA,* GMRα, or CD116*)* is one of the receptors for granulocyte macrophage-colony stimulating factor (GM-CSF). The GM-CSF receptor is an oligomer of the alpha and beta (*CSF2RB*, GMRβ, or CD131) receptors (Figure 5). The alpha subunit binds with high affinity to GM-CSF and confers specificity of signaling. The beta subunit of GM-CSF also serves as the beta receptor for the IL-3, as aforementioned, and IL-5 (Interleukin 5) receptors, which both have unique alpha receptors that provide signaling specificity [59]. GM-CSF signaling regulates various cellular functions including cell proliferation, survival, and differentiation. Increased levels of *CSF2RA* and GM-CSF signaling have been observed in hematologic malignancies such as CMML (chronic myelomonocytic leukemia) and JMML (juvenile myelomonocytic leukemia) [60, 61]. Leukemia cells from JMML and CMML also frequently exhibit hypersensitivity to GM and harbor oncogenic RAS mutations, which results in continuous activation of the RAS-RAF-MEK-ERK and JAK2-STAT5 signaling pathways [60, 62]. Interestingly, treatment of these cells with the JAK2 inhibitor XL019 inhibited both STAT5 and ERK phosphorylation in the presence of GM-CSF; however, treatment with the MEK inhibitor CI-1040 failed to inhibit STAT5 activation in the presence of GM-CSF [62]. These observations suggest that GM-CSF may be activating JAK2-STAT5 in a RAS- and RAS effector-independent fashion. Studies with an *Nras*^G12D/+^ driven mouse model for CMML demonstrated that eliminating GM-CSF signaling via knockout of the GM-CSF beta receptor was insufficient to inhibit leukemia development, but prolonged the survival of mice and reduced splenomegaly of NRas^G12D/+^ mice, and reduced colony formation of their hematopoietic cells [63]. These findings suggest that GM-CSF signaling may cooperate to provide mitogenic signals in CMML and JMML, but indicates there are other oncogenic drivers responsible for leukemic transformation.

Conversely, LOH of *CSF2RA*, along with *SHOX* and *CRLF2*, due to deletion in a portion of the PAR has been reported in mantle cell lymphoma [28]. As mentioned previously, the t(8;21) requires additional cooperating mutations for leukemogenesis, and roughly 32-59% of t(8;21) patients also display LOS [21, 22], suggesting that genes on the sex chromosomes, such as *CSF2RA*, may act as tumor suppressors. In fact, loss of GM-CSF signaling cooperated with RUNX1-ETO, the oncofusion protein generated from t(8;21), to induce AML in a murine model [64]. Together, these data support that GM-CSF signaling has negative effects in t(8;21) AML development, and substantiates that reduced *CSF2RA* levels due to LOS may cooperate with t(8;21) in leukemogenesis.

#### P2RY8

Another PAR gene with context-dependent functions is *P2RY8*. This gene encodes the Purinergic receptor P2Y8 (*P2RY8*), an orphan receptor that is a member of the G-protein coupled receptor family. This family of receptors is activated upon binding of adenosine and uracil nucleotides. *P2RY8* is highly expressed in hematopoietic cells, including granulocyte-macrophage progenitor cells, B cells, and T cells [44]. Additionally, *P2RY8* has been found to be upregulated in leukemias and expression of P2RY8 in 3T3 cells resulted in their ability to form tumors *in vivo*, suggesting it may serve as a proto-oncogene [65].

Contrarily, loss of function mutations of *P2RY8* are frequently detected in Burkitt's lymphoma and lymphomas derived from germinal center B cells. Five of the six mutations identified in one study resulted in loss of P2RY8 expression at the cell surface [66]. Additionally, ectopic expression of P2RY8 was demonstrated to suppress germinal center B cell proliferation. Therefore, P2RY8 may aid in suppressing B cell proliferation. In the case of the aforementioned *P2RY8*-*CRLF2* rearrangement, where the coding sequence of *P2RY8* is deleted, it may consequently result in deletion of a potential tumor suppressor [49] (Figure 4). Given that the regulatory element of *P2RY8* is highly active in lymphoid cells, it is fitting that the P2RY8-CRLF2 rearrangement, which places the proto-oncogenic *CRLF2* under control of the *P2RY8* regulatory element, is correlated with lymphoid leukemia.

#### IL9R

Interleukin 9 Receptor (*IL9R* or CD129) heterodimerizes with interleukin 2 receptor gamma (IL2R) to form the receptor for its ligand interleukin 9 (IL9). IL9 is a cytokine that stimulates proliferation and inhibits apoptosis. It is also a growth factor for several hematological cell types, including T cells, mast cells, and leukemia cell lines. IL9R is most highly expressed on T cells, and has been found to be upregulated in various hematological malignancies, including B and T cell leukemias, B cell lymphomas, and AML [44]. Although IL9R generally forms heterodimers with IL2R, IL9R has also been found to form homodimers that interact with and activate mutant JAK1, which is common in ALL, to further activate its downstream STAT targets [67]. Contrarily, IL9 has been reported to activate tumor immunity. In a pulmonary melanoma model, it was found that neutralization of IL9 resulted in increased tumor growth and reduced leukocyte infiltration in the tumors [68]. In another study of mice bearing B16F10 melanoma, homozygous knockout of *IL9RA* resulted in increased tumor growth, and administration of IL9 to wildtype tumor-bearing mice reduced tumor growth [69]. Altogether, these findings suggest that IL9 signaling promotes activation of immune cells to recognize cancer cells and has tumor suppressive functions. In hematopoietic cells with LOS, reduced *IL9R* levels could hinder activation of an antitumor response, thus allowing cancer cells to escape from immunosurveillance.

#### CD99

*CD99* (*MIC2*, *HBA71*, *MSK5X*), also known as the E2 antigen, is a glycoprotein that is expressed across the hematopoietic system and decreases with differentiation. CD99 is most highly expressed on thymocytes where it regulates T-cell adhesion and apoptosis [70]. CD99 has been found to be upregulated in acute lymphoblastic leukemias (ALL) [70, 71]. Incubation with CD99 antibody has been reported to trigger apoptosis of the Jurkat cell line, thymocytes, TEL/AML1-positive ALL cells, and normal B cell precusors [71, 72]. Additionally, binding of specific epitopes of CD99 has been found to promote cell death of transformed T cells via a caspase-independent pathway [73]. Although CD99 overexpression is observed in various hematologic malignancies, *CD99* haploinsufficiency could also perturb normal T cell apoptosis and cell adhesion and result in increased T cell survival and mobility.

The conflicting findings for these PAR genes stress the importance of cellular context when studying the functions of PAR genes in hematological malignancies.

### Inactive PAR genes

Although most PAR genes escape inactivation, two PAR genes, *SPRY3* and *VAMP7*, are silenced on the inactive X chromosome in females and the Y chromosome in males, which results in monoallelic expression. The mechanisms of inactivation of the two genes vary. Treatment of cells with DNA methylation inhibitors enhanced *VAMP7*, but not *SPRY3* expression, suggesting that promoter methylation is responsible for inactivating *VAMP7*, but not *SPRY3* [74, 75]. Instead, histone modifications appear to be responsible for the maintenance of *SPRY3* repression, as treatment with histone deacetylase (HDAC) inhibitors reactivated its expression [75]. *SPRY3* (sprouty RTK signaling antagonist 3) encodes one of the four mammalian Sprouty proteins (SPRY1-4), which were originally identified in *Drosophila* to function as inhibitors of MAPK signaling [76]. However, very little is known about SPRY3. Because *SPRY3* is a monoallelically expressed PAR gene, its expression should not be affected by LOS.

Vesicle associated membrane protein 7 (*VAMP7, SYBL1, TIVAMP*) is found in the membranes of late endosomes and lysosomes. VAMP7 is a member of the soluble NSF attachment protein receptor (SNARE) family. VAMP7 aids in the fusion of vesicles to their target membranes and is required for the formation of mature autophagosomes [77]. Knockdown of VAMP7 reduced autophagosome formation in starvation conditions and resulted in an accumulation of autophagosomes due to impaired ability to fuse to lysosomes [78]. VAMP7 has also been reported to be required for the release of cytotoxic granules in natural killer (NK) cells [79], and controls T cell activation by regulating the transport and docking of vesicles containing critical adaptor proteins for T cell activation to T cell receptor-activation sites [80]. *VAMP7* has various functions that could be regarded as tumor suppressive; therefore, aberrant epigenetic silencing of the active allele could be involved in cellular transformation.

### Other less characterized PAR genes and regions

As the remaining PAR genes are not as well characterized, there exists great potential for novel functional studies of these genes in hematopoietic development and diseases. In a recent report attempting to identify tumor suppressors in malignant melanoma, *PLCXD1* (Phosphatidylinositol-specific phospholipase C, X domain containing 1) was one of 7 genes to be methylated or deleted in all 14 melanoma tumor samples that were examined [81]. Additionally, when *PLCXD1* was ectopically expressed, it reduced proliferation in 2 of the 4 melanoma cell lines that were assessed [81]. Unfortunately, *PLCXD1* remains a relatively uncharacterized gene.

*ASMTL* is expressed highly in T lymphocytes [44] and codes for the N-acetylserotonin O-methyltransferase-like protein. ASMTL has two domains with homology to two different proteins. The N-terminal domain shares roughly 60% sequence homology to two bacterial proteins: maf of *B. subtilis* and orfE of *E. Coli*. The C-terminal domain is highly homologous to ASMT (acetylserotonin O-methyltransferase), which is encoded by another PAR gene [82]. Very little has been reported on the function of ASMTL. However, in a genomic study of high risk childhood B-cell precursor ALL, recurrent point mutations of *ASMTL* were discovered [83]. One of the identified mutations results in a frameshift mutation at V171, and the other results in an R395H missense mutation.

A-kinase anchoring protein 17A (*AKAP17A, XE7, CCDC133, PRKA17A, SFRS17A, AKAP-17A, DXYS155E, CXYorf3*) is highly expressed in T cells, B cells, and dendritic cells [44]. AKAP17A is a protein kinase A anchoring protein (AKAP) and binds to both type I and II PKA to localize it to subcellular regions [84]. In addition to binding PKA, AKAP17A has also been found to interact with the splicing factors ZNF265, ASF/SF2, and SC35, and therefore acts as a regulator of alternative splicing, which is often perturbed in cancer cells [84, 85].

Zinc finger BED-containing 1 (*ZBED1*, *ALTE*, *DREF*, *TRAMP*) is a transcription factor that regulates DNA replication and cell proliferation genes [86, 87]. There have been no studies of *ZBED1* in the hematopoietic system. However, knockdown of *ZBED1/DREF* in HeLa cells reduced cell proliferation and reduced expression of its target gene *FNC16* (histone H1) [87].

*XG* or *PBDX* encodes a blood group antigen that shares 48% sequence homology with CD99 [88]. XG expression is believed to be restricted to red blood cells, and cells are either Xg(a+) or Xg(a-). In red blood cells of those who are Xg(a-), no CD99 was detected, suggesting that expression of *XG* is co-regulated with *CD99* [89]. Further studies confirmed that the expression of these two genes are in fact co-regulated, but only at the transcriptional level [90, 91]. Aside from encoding a blood cell antigen, there are no other reported functions of this gene.

GTPases have been demonstrated to regulate hematopoietic stem cell functions and differentiation, and both activating and inactivating mutations have been reported in hematologic diseases [92]. *GTPBP6* (*PGPL*) encodes a protein that is 442 amino acids long. Because it contains 4 characteristic GTP-binding sites, it has been characterized as a putative GTP-binding protein [93]. Although *GTPBP6* is highly expressed in CD4 T lymphocytes, myoblasts, and erythroid cells, there have been no studies of *GTPBP6* in the hematopoietic system [44]. Given the importance of GTP-binding proteins in regulating cellular functions, additional mechanistic studies on the functions of *GTPBP6* in normal and malignant hematopoietic contexts would be of great value.

In addition to protein coding genes, there are several uncharacterized long intergenic non-protein coding RNAs, including LINC00106, LINC00685, and LINC00102, and the pseudogene *DDX11L16* (DEAD/H box polypeptide 11 like 16), in the PARs [94, 95]. There are no validated microRNAs (miRNAs) in the PARs.

## SEX CHROMOSOME AMPLIFICATIONS IN HEMATOLOGICAL MALIGNANCIES

Although in this review we focus on LOS, sex chromosome amplifications have also been reported in hematological malignancies. Extra copies of the X and Y chromosomes have been detected in chronic neutrophilic leukemia (CNL) that progressed to myeloid blast crisis [96] and AML [97]. Gain of an X chromosome (+X) is a frequent finding in both childhood and adult ALL patients [98–100]. One of the most common cytogenetic abnormalities associated with childhood B-cell ALL is hyperdiploidy, and close to 95% of these patients display gain of an additional X chromosome [99]. Gain of an X chromosome is also significantly overrepresented in patients with down-syndrome-associated ALL (DS-ALL) [100]. Half of DS-ALL patients with +X exhibit +X as the sole additional chromosomal abnormality, suggesting that gain of an X chromosome can synergize with trisomy 21 to promote lymphoid malignancy [100]. In addition, men with Klinefelter syndrome (XXY) were found to have increased risk to develop non-Hodgkin lymphoma [101, 102]. Hypodiploidy is also a common cytogenetic abnormality in ALL patients, however LOS (X or Y) in this group is rare, suggesting that sex chromosome genes may not play a tumor suppressive role in the context of ALL development [103].

Given that some PAR genes do possess potentially oncogenic functions, such as promoting cell proliferation, it is not surprising that sex chromosome polysomies are also observed in hematological malignancies and their roles in oncogenesis should not be overlooked.

## MODELS FOR LOS

There are several mouse models available for modeling LOS. One such model is that for Turner Syndrome, which is denoted as XO or 39, XO, where mice have only a single X chromosome with an X-linked marker so that the sex chromosome status can be easily distinguished by fur coat color [104]. Another mouse model for LOS is the X-linked patchy fur (Paf) mutant mouse, which results in 19% of female offspring from *Paf* mutant males being XO [105]. These mice were further modified to carry a variant Y chromosome (Y*) that increased the rate of XO female offspring to roughly 40% [106].

While LOS mouse models exist, they are inadequate at fully recapitulating the effects of human LOS due to divergence of the PARs in mice and humans. Therefore, for *in vivo* studies of PAR genes in cancer development and progression, it is critical to consider the differences in the genetic composition of human and mice PARs.

Many human PAR genes are not located on the PARs in mice or do not possess a murine homolog (Table 2). For example, the human PAR1 genes *CSF2RA* and *IL3RA* are located on murine chromosomes 19, and 14, respectively [107]. The human PAR2 gene *IL9R* is located on murine chromosome 11. There also exist genes in murine PARs that are not located in human PARs. Altogether, these differences in the human and murine PARs illustrate the complications with LOS mouse models and demonstrate the importance of examining PAR genes on an individual basis in future models. Further supporting the need to study individual PAR genes, as opposed to gross sex chromosomal loss, is the inability to exclude the involvement of other genomic loci on the sex chromosomes in murine LOS models.

**Table 2:** Human PAR genes and their location in the murine genome

Human PAR Gene		Location in mouse genome
PAR1 Genes	*PLCXD1*	Chromosome 5
	*GTPBP6*	Chromosome 5
	*PPP2R3B*	Absent in mice
	*SHOX*	Absent in mice
	*CRLF2*	Chromosome 5
	*CSF2RA*	Chromosome 19
	*IL3RA*	Chromosome 14
	*SLC25A6*	Chromosome 6
	*ASMTL*	Absent in mice
	*P2RY8*	Absent in mice
	*AKAP17A*	Absent in mice
	*DHRSX*	Chromosome 4 (Unlocalized scaffold)
	*ZBED1*	Absent in mice
	*CD99*	Chromosome 4 (Unlocalized scaffold)
	*XG*	Absent in mice
PAR2 Genes	*SPRY3*	X Chromosome (Unlocalized scaffold)
	*VAMP7*	X Chromosome (Unlocalized scaffold)
	*IL9R*	Chromosome 11

The PAR genes' involvement in a wide breadth of cellular functions, some of which are potentially tumor suppressive or oncogenic, highlights the complexity of studying the involvement of LOS and PAR genes in tumorigenesis. The seemingly contradictory functions reported for various PAR genes emphasize the importance of cellular context when studying the roles of LOS and PAR genes in the development of hematologic malignancies and other cancers.

Therefore, targeted disruption of individual PAR genes, or a combination of PAR genes, remains the most definitive method to characterize the roles of these genes in cancer models. With the advent of CRISPR-Cas genome editing, disruption, knockout, and mutation of specific genes has become more feasible and efficient. Additionally, the CRISPR-Cas system allows for the efficient generation of genetically modified mice with alterations in multiple genes, which is immensely useful for studying cooperating mutations [108]. Consequently, instead of relying on mouse LOS models with chromosomal features similar to human LOS, mouse models with disruption or alteration of individual or several PAR genes can now be generated for further mechanistic studies. Additionally, for human PAR genes without murine homologs, CRISPR-Cas-mediated alteration of those genes in human cells can be used in xenograft murine models and would serve as an alternative method to study these genes *in vivo*.

## FUTURE DIRECTIONS

Given the high frequency of LOS in hematological malignancies and the shortage of systematic studies of PAR genes in the hematopoietic system, there remains the potential to uncover novel functions for PAR genes, which could be targeted therapeutically. However, in addition to the PAR genes, over 100 X chromosome genes have been reported to escape X-inactivation and are also expressed biallelically [109, 110]. Therefore, upon LOS, these genes also suffer haploinsufficiency. Further mechanistic studies to unravel potential tumor suppressive function of the genes that escape X-inactivation would provide additional insight into how LOS may contribute to hematological malignancies.

Being that several PAR genes are implicated to function as tumor suppressors, such as *PPP2R3B*, *SHOX*, *SLC25A6*, *ASMT*, and *DHRSX*, they serve as prime candidates for further investigation and may hold the greatest therapeutic promise. For example, if haploinsufficiency of *PPP2R3B*, a subunit of PP2A, aids in the development of hematological malignancies, this could expand the applications for pharmacological activators of PP2A, which are being explored in preclinical AML models [111]. Furthermore, if reduced levels of melatonin or *ASMT*, which catalyzes the final step in melatonin synthesis, contributes to tumorigenesis, it would be worthwhile to explore the effects of supplementing current treatments with melatonin. Importantly, elucidating the role of haploinsufficiency of PAR genes in the development of hematological malignancies and chemoresistance could reveal a novel function for LOS as a predictive biomarker for patient prognoses and therapeutic response. Studies clarifying the mechanisms of action of PAR genes may also provide valuable insight into viable combination therapies that would be especially beneficial for patients exhibiting LOS compared to those who do not. Altogether, mechanistic studies on the effects of LOS and haploinsufficiency of PAR genes are currently lacking and may greatly assist in opening the door to alternative therapeutic strategies for patients suffering from hematological malignancies.

## References

[1] Fröhling S, Döhner H (2008). Chromosomal abnormalities in cancer. N Engl J Med.

[2] Korkmaz DT, Demirhan O, Abat D, Demirberk B, Tunç E, Kuleci S (2015). Microchimeric Cells, Sex Chromosome Aneuploidies and Cancer. Pathol Oncol Res.

[3] Missiaglia E, Moore PS, Williamson J, Lemoine NR, Falconi M, Zamboni G, Scarpa A (2002). Sex chromosome anomalies in pancreatic endocrine tumors. Int J Cancer.

[4] Jacobs PA, Maloney V, Cooke R, Crolla JA, Ashworth A, Swerdlow AJ (2013). Male breast cancer, age and sex chromosome aneuploidy. Br J Cancer.

[5] Borah V, Shah PN, Ghosh SN, Sampat MB, Jussawalla DJ (1980). Further studies on the prognostic importance of Barr body frequency in human breast cancer: with discussion on its probable mechanism. J Surg Oncol.

[6] Bottarelli L, Azzoni C, Necchi F, Lagrasta C, Tamburini E, D'Adda T, Pizzi S, Sarli L, Rindi G, Bordi C (2007). Sex chromosome alterations associate with tumor progression in sporadic colorectal carcinomas. Clin Cancer Res.

[7] Cantú ES, Moses MD, Nemana LJ, Pierre RV (2015). Sex chromosome loss in adults with haematological neoplasms. Br J Haematol.

[8] Huh J, Moon H, Chung WS (2007). [Incidence and clinical significance of sex chromosome losses in bone marrow of patients with hematologic diseases]. Korean J Lab Med [Article in Korean].

[9] Wong AK, Fang B, Zhang L, Guo X, Lee S, Schreck R (2008). Loss of the Y chromosome: an age-related or clonal phenomenon in acute myelogenous leukemia/myelodysplastic syndrome?. Arch Pathol Lab Med.

[10] Stone JF, Sandberg AA (1995). Sex chromosome aneuploidy and aging. Mutat Res.

[11] Guttenbach M, Koschorz B, Bernthaler U, Grimm T, Schmid M (1995). Sex chromosome loss and aging: in situ hybridization studies on human interphase nuclei. Am J Hum Genet.

[12] Pierre RV, Hoagland HC (1972). Age-associated aneuploidy: loss of Y chromosome from human bone marrow cells with aging. Cancer.

[13] Jacobs PA, Court Brown WM, Doll R (1961). Distribution of human chromosome counts in relation to age. Nature.

[14] Sandberg AA, Cohen MM, Rimm AA, Levin ML (1967). Aneuploidy and age in a population survey. Am J Hum Genet.

[15] Hando JC, Tucker JD, Davenport M, Tepperberg J, Nath J (1997). X chromosome inactivation and micronuclei in normal and Turner individuals. Hum Genet.

[16] Riesch M, Niggli FK, Leibundgut K, Caflisch U, Betts DR (2001). Loss of X chromosome in childhood acute lymphoblastic leukemia. Cancer Genet Cytogenet.

[17] Heerema NA, Nachman JB, Sather HN, Sensel MG, Lee MK, Hutchinson R, Lange BJ, Steinherz PG, Bostrom B, Gaynon PS, Uckun F (1999). Hypodiploidy with less than 45 chromosomes confers adverse risk in childhood acute lymphoblastic leukemia: a report from the children's cancer group. Blood.

[18] Bakshi S, Kakadia P, Brahmbhatt M, Trivedi P, Rawal S, Bhatt S, Parikh B, Patel K, Shukla S, Shah P (2004). Loss of sex chromosome in acute myeloid leukemia.

[19] Chapiro E, Antony-Debre I, Marchay N, Parizot C, Lesty C, Cung HA, Mathis S, Grelier A, Maloum K, Choquet S, Azgui Z, Uzunov M, Leblond V (2014). Sex chromosome loss may represent a disease-associated clonal population in chronic lymphocytic leukemia. Genes Chromosomes Cancer.

[20] Manola KN, Sambani C, Karakasis D, Kalliakosta G, Harhalakis N, Papaioannou M (2008). Leukemias associated with Turner syndrome: report of three cases and review of the literature. Leuk Res.

[21] Kuchenbauer F, Schnittger S, Look T, Gilliland G, Tenen D, Haferlach T, Hiddemann W, Buske C, Schoch C (2006). Identification of additional cytogenetic and molecular genetic abnormalities in acute myeloid leukaemia with t(8;21)/AML1-ETO. Br J Haematol.

[22] Marcucci G, Mrozek K, Ruppert AS, Maharry K, Kolitz JE, Moore JO, Mayer RJ, Pettenati MJ, Powell BL, Edwards CG, Sterling LJ, Vardiman JW, Schiffer CA (2005). Prognostic factors and outcome of core binding factor acute myeloid leukemia patients with t(8;21) differ from those of patients with inv(16): a Cancer and Leukemia Group B study. J Clin Oncol.

[23] Yuan Y, Zhou L, Miyamoto T, Iwasaki H, Harakawa N, Hetherington CJ, Burel SA, Lagasse E, Weissman IL, Akashi K, Zhang DE (2001). AML1-ETO expression is directly involved in the development of acute myeloid leukemia in the presence of additional mutations. Proc Natl Acad Sci U S A.

[24] Briggs SF, Reijo Pera RA (2014). X chromosome inactivation: recent advances and a look forward. Curr Opin Genet Dev.

[25] Rappold GA (1993). The pseudoautosomal regions of the human sex chromosomes. Hum Genet.

[26] Helena Mangs A, Morris BJ (2007). The Human Pseudoautosomal Region (PAR): Origin, Function and Future. Curr Genomics.

[27] Veerappa AM, Padakannaya P, Ramachandra NB (2013). Copy number variation-based polymorphism in a new pseudoautosomal region 3 (PAR3) of a human X-chromosome-transposed region (XTR) in the Y chromosome. Funct Integr Genomics.

[28] Nieländer I, Martín-Subero JI, Wagner F, Baudis M, Gesk S, Harder L, Hasenclever D, Klapper W, Kreuz M, Pott C, Martinez-Climent JA, Dreyling M, Arnold N (2008). Recurrent loss of the Y chromosome and homozygous deletions within the pseudoautosomal region 1: association with male predominance in mantle cell lymphoma. Haematologica.

[29] Cerami E, Gao J, Dogrusoz U, Gross BE, Sumer SO, Aksoy BA, Jacobsen A, Byrne CJ, Heuer ML, Larsson E, Antipin Y, Reva B, Goldberg AP (2012). The cBio cancer genomics portal: an open platform for exploring multidimensional cancer genomics data. Cancer Discov.

[30] Mumby M (2007). PP2A: unveiling a reluctant tumor suppressor. Cell.

[31] Perrotti D, Jamieson C, Goldman J, Skorski T (2010). Chronic myeloid leukemia: mechanisms of blastic transformation. J Clin Invest.

[32] Neviani P, Harb JG, Oaks JJ, Santhanam R, Walker CJ, Ellis JJ, Ferenchak G, Dorrance AM, Paisie CA, Eiring AM, Ma Y, Mao HC, Zhang B (2013). PP2A-activating drugs selectively eradicate TKI-resistant chronic myeloid leukemic stem cells. J Clin Invest.

[33] Yang Y, Huang Q, Lu Y, Li X, Huang S (2012). Reactivating PP2A by FTY720 as a novel therapy for AML with C-KIT tyrosine kinase domain mutation. J Cell Biochem.

[34] Ramaswamy K, Spitzer B, Kentsis A (2015). Therapeutic Re-Activation of Protein Phosphatase 2A in Acute Myeloid Leukemia. Front Oncol.

[35] Yang Z, Cheng W, Hong L, Chen W, Wang Y, Lin S, Han J, Zhou H, Gu J (2007). Adenine nucleotide (ADP/ATP) translocase 3 participates in the tumor necrosis factor induced apoptosis of MCF-7 cells. Mol Biol Cell.

[36] Zamora M, Ortega JA, Alaña L, Viñas O, Mampel T (2006). Apoptotic and anti-proliferative effects of all-trans retinoic acid. Adenine nucleotide translocase sensitizes HeLa cells to all-trans retinoic acid. Exp Cell Res.

[37] Hu Z, Guo X, Yu Q, Qiu L, Li J, Ying K, Guo C, Zhang J (2009). Down-regulation of adenine nucleotide translocase 3 and its role in camptothecin-induced apoptosis in human hepatoma QGY7703 cells. FEBS Lett.

[38] Altieri A, Hemminki K (2006). The familial risk of Hodgkin's lymphoma ranks among the highest in the Swedish Family-Cancer Database. Leukemia.

[39] Grufferman S, Cole P, Smith PG, Lukes RJ (1977). Hodgkin's disease in siblings. N Engl J Med.

[40] Horwitz MS, Mealiffe ME (2007). Further evidence for a pseudoautosomal gene for Hodgkin's lymphoma: Reply to ‘The familial risk of Hodgkin's lymphoma ranks among the highest in the Swedish Family-Cancer Database' by Altieri A and Hemminki K. Leukemia.

[41] Büyükavci M, Ozdemir O, Buck S, Stout M, Ravindranath Y, Savaşan S (2006). Melatonin cytotoxicity in human leukemia cells: relation with its pro-oxidant effect. Fundam Clin Pharmacol.

[42] Perdomo J, Cabrera J, Estévez F, Loro J, Reiter RJ, Quintana J (2013). Melatonin induces apoptosis through a caspase-dependent but reactive oxygen species-independent mechanism in human leukemia Molt-3 cells. J Pineal Res.

[43] Conti A, Conconi S, Hertens E, Skwarlo-Sonta K, Markowska M, Maestroni JM (2000). Evidence for melatonin synthesis in mouse and human bone marrow cells. J Pineal Res.

[44] Hruz T, Laule O, Szabo G, Wessendorp F, Bleuler S, Oertle L, Widmayer P, Gruissem W, Zimmermann P (2008). Genevestigator v3: a reference expression database for the meta-analysis of transcriptomes. Adv Bioinformatics.

[45] Yamanishi M, Narazaki H, Asano T (2015). Melatonin overcomes resistance to clofarabine in two leukemic cell lines by increased expression of deoxycytidine kinase. Exp Hematol.

[46] Sehgal AR, Konig H, Johnson DE, Tang D, Amaravadi RK, Boyiadzis M, Lotze MT (2015). You eat what you are: autophagy inhibition as a therapeutic strategy in leukemia. Leukemia.

[47] Zhang G, Luo Y, Li G, Wang L, Na D, Wu X, Zhang Y, Mo X (2014). DHRSX, a novel non-classical secretory protein associated with starvation induced autophagy. Int J Med Sci.

[48] Rochman Y, Kashyap M, Robinson GW, Sakamoto K, Gomez-Rodriguez J, Wagner KU, Leonard WJ (2010). Thymic stromal lymphopoietin-mediated STAT5 phosphorylation via kinases JAK1 and JAK2 reveals a key difference from IL-7-induced signaling. Proc Natl Acad Sci U S A.

[49] Hertzberg L, Vendramini E, Ganmore I, Cazzaniga G, Schmitz M, Chalker J, Shiloh R, Iacobucci I, Shochat C, Zeligson S, Cario G, Stanulla M, Strehl S (2010). Down syndrome acute lymphoblastic leukemia, a highly heterogeneous disease in which aberrant expression of CRLF2 is associated with mutated JAK2: a report from the International BFM Study Group. Blood.

[50] Mullighan CG, Collins-Underwood JR, Phillips LA, Loudin MG, Liu W, Zhang J, Ma J, Coustan-Smith E, Harvey RC, Willman CL, Mikhail FM, Meyer J, Carroll AJ (2009). Rearrangement of CRLF2 in B-progenitor- and Down syndrome-associated acute lymphoblastic leukemia. Nat Genet.

[51] Russell LJ, Capasso M, Vater I, Akasaka T, Bernard OA, Calasanz MJ, Chandrasekaran T, Chapiro E, Gesk S, Griffiths M, Guttery DS, Haferlach C, Harder L (2009). Deregulated expression of cytokine receptor gene, CRLF2, is involved in lymphoid transformation in B-cell precursor acute lymphoblastic leukemia. Blood.

[52] Tasian SK, Loh ML (2011). Understanding the biology of CRLF2-overexpressing acute lymphoblastic leukemia. Crit Rev Oncog.

[53] Testa U, Riccioni R, Diverio D, Rossini A, Lo Coco F, Peschle C (2004). Interleukin-3 receptor in acute leukemia. Leukemia.

[54] Muñoz L, Nomdedéu JF, López O, Carnicer MJ, Bellido M, Aventín A, Brunet S, Sierra J (2001). Interleukin-3 receptor alpha chain (CD123) is widely expressed in hematologic malignancies. Haematologica.

[55] Testa U, Riccioni R, Militi S, Coccia E, Stellacci E, Samoggia P, Latagliata R, Mariani G, Rossini A, Battistini A, Lo-Coco F, Peschle C (2002). Elevated expression of IL-3Ralpha in acute myelogenous leukemia is associated with enhanced blast proliferation, increased cellularity, and poor prognosis. Blood.

[56] Busfield SJ, Biondo M, Wong M, Ramshaw HS, Lee EM, Ghosh S, Braley H, Panousis C, Roberts AW, He SZ, Thomas D, Fabri L, Vairo G (2014). Targeting of acute myeloid leukemia in vitro and in vivo with an anti-CD123 mAb engineered for optimal ADCC. Leukemia.

[57] Pizzitola I, Anjos-Afonso F, Rouault-Pierre K, Lassailly F, Tettamanti S, Spinelli O, Biondi A, Biagi E, Bonnet D (2014). Chimeric antigen receptors against CD33/CD123 antigens efficiently target primary acute myeloid leukemia cells in vivo. Leukemia.

[58] Mardiros A, Dos Santos C, McDonald T, Brown CE, Wang X, Budde LE, Hoffman L, Aguilar B, Chang WC, Bretzlaff W, Chang B, Jonnalagadda M, Starr R (2013). T cells expressing CD123-specific chimeric antigen receptors exhibit specific cytolytic effector functions and antitumor effects against human acute myeloid leukemia. Blood.

[59] Hansen G, Hercus TR, McClure BJ, Stomski FC, Dottore M, Powell J, Ramshaw H, Woodcock JM, Xu Y, Guthridge M, McKinstry WJ, Lopez AF, Parker MW (2008). The structure of the GM-CSF receptor complex reveals a distinct mode of cytokine receptor activation. Cell.

[60] de Vries AC, Zwaan CM, van den Heuvel-Eibrink MM (2010). Molecular basis of juvenile myelomonocytic leukemia. Haematologica.

[61] Padron E, Painter JS, Kunigal S, Mailloux AW, McGraw K, McDaniel JM, Kim E, Bebbington C, Baer M, Yarranton G, Lancet J, Komrokji RS, Abdel-Wahab O (2013). GM-CSF-dependent pSTAT5 sensitivity is a feature with therapeutic potential in chronic myelomonocytic leukemia. Blood.

[62] Kotecha N, Flores NJ, Irish JM, Simonds EF, Sakai DS, Archambeault S, Diaz-Flores E, Coram M, Shannon KM, Nolan GP, Loh ML (2008). Single-cell profiling identifies aberrant STAT5 activation in myeloid malignancies with specific clinical and biologic correlates. Cancer Cell.

[63] Zhang J, Ranheim EA, Du J, Liu Y, Wang J, Kong G (2015). Deficiency of beta common receptor moderately attenuates the progression of myeloproliferative neoplasm in Nras G12D/+ mice. J Biol Chem.

[64] Matsuura S, Yan M, Lo MC, Ahn EY, Weng S, Dangoor D, Matin M, Higashi T, Feng GS, Zhang DE (2012). Negative effects of GM-CSF signaling in a murine model of t(8;21)-induced leukemia. Blood.

[65] Fujiwara S, Yamashita Y, Choi YL, Watanabe H, Kurashina K, Soda M, Enomoto M, Hatanaka H, Takada S, Ozawa K, Mano H (2007). Transforming activity of purinergic receptor P2Y, G protein coupled, 8 revealed by retroviral expression screening. Leuk Lymphoma.

[66] Muppidi JR, Schmitz R, Green JA, Xiao W, Larsen AB, Braun SE, An J, Xu Y, Rosenwald A, Ott G, Gascoyne RD, Rimsza LM, Campo E (2014). Loss of signalling via Gα13 in germinal centre B-cell-derived lymphoma. Nature.

[67] Hornakova T, Staerk J, Royer Y, Flex E, Tartaglia M, Constantinescu SN, Knoops L, Renauld JC (2009). Acute lymphoblastic leukemia-associated JAK1 mutants activate the Janus kinase/STAT pathway via interleukin-9 receptor alpha homodimers. J Biol Chem.

[68] Lu Y, Hong S, Li H, Park J, Hong B, Wang L, Zheng Y, Liu Z, Xu J, He J, Yang J, Qian J, Yi Q (2012). Th9 cells promote antitumor immune responses in vivo. J Clin Invest.

[69] Purwar R, Schlapbach C, Xiao S, Kang HS, Elyaman W, Jiang X, Jetten AM, Khoury SJ, Fuhlbrigge RC, Kuchroo VK, Clark RA, Kupper TS (2012). Robust tumor immunity to melanoma mediated by interleukin-9-producing T cells. Nat Med.

[70] Dworzak MN, Fröschl G, Printz D, Zen LD, Gaipa G, Ratei R, Basso G, Biondi A, Ludwig WD, Gadner H (2004). CD99 expression in T-lineage ALL: implications for flow cytometric detection of minimal residual disease. Leukemia.

[71] Husak Z, Printz D, Schumich A, Pötschger U, Dworzak MN (2010). Death induction by CD99 ligation in TEL/AML1-positive acute lymphoblastic leukemia and normal B cell precursors. J Leukoc Biol.

[72] Bernard G, Breittmayer JP, de Matteis M, Trampont P, Hofman P, Senik A, Bernard A (1997). Apoptosis of immature thymocytes mediated by E2/CD99. J Immunol.

[73] Pettersen RD, Bernard G, Olafsen MK, Pourtein M, Lie SO (2001). CD99 signals caspase-independent T cell death. J Immunol.

[74] Matarazzo MR, De Bonis ML, Gregory RI, Vacca M, Hansen RS, Mercadante G, D'Urso M, Feil R, D'Esposito M (2002). Allelic inactivation of the pseudoautosomal gene SYBL1 is controlled by epigenetic mechanisms common to the X and Y chromosomes. Hum Mol Genet.

[75] De Bonis ML, Cerase A, Matarazzo MR, Ferraro M, Strazzullo M, Hansen RS, Chiurazzi P, Neri G, D'Esposito M (2006). Maintenance of X- and Y-inactivation of the pseudoautosomal (PAR2) gene SPRY3 is independent from DNA methylation and associated to multiple layers of epigenetic modifications. Hum Mol Genet.

[76] Hacohen N, Kramer S, Sutherland D, Hiromi Y, Krasnow MA (1998). sprouty encodes a novel antagonist of FGF signaling that patterns apical branching of the Drosophila airways. Cell.

[77] Moreau K, Rubinsztein DC (2012). The plasma membrane as a control center for autophagy. Autophagy.

[78] Moreau K, Ravikumar B, Renna M, Puri C, Rubinsztein DC (2011). Autophagosome precursor maturation requires homotypic fusion. Cell.

[79] Krzewski K, Gil-Krzewska A, Watts J, Stern JN, Strominger JL (2011). VAMP4- and VAMP7-expressing vesicles are both required for cytotoxic granule exocytosis in NK cells. Eur J Immunol.

[80] Larghi P, Williamson DJ, Carpier JM, Dogniaux S, Chemin K, Bohineust A, Danglot L, Gaus K, Galli T, Hivroz C (2013). VAMP7 controls T cell activation by regulating the recruitment and phosphorylation of vesicular Lat at TCR-activation sites. Nat Immunol.

[81] Mithani SK, Smith IM, Califano JA (2011). Use of integrative epigenetic and cytogenetic analyses to identify novel tumor-suppressor genes in malignant melanoma. Melanoma Res.

[82] Ried K, Rao E, Schiebel K, Rappold GA (1998). Gene duplications as a recurrent theme in the evolution of the human pseudoautosomal region 1: isolation of the gene ASMTL. Hum Mol Genet.

[83] Zhang J, Mullighan CG, Harvey RC, Wu G, Chen X, Edmonson M, Buetow KH, Carroll WL, Chen IM, Devidas M, Gerhard DS, Loh ML, Reaman GH (2011). Key pathways are frequently mutated in high-risk childhood acute lymphoblastic leukemia: a report from the Children's Oncology Group. Blood.

[84] Jarnaess E, Stokka AJ, Kvissel AK, Skålhegg BS, Torgersen KM, Scott JD, Carlson CR, Taskén K (2009). Splicing factor arginine/serine-rich 17A (SFRS17A) is an A-kinase anchoring protein that targets protein kinase A to splicing factor compartments. J Biol Chem.

[85] Mangs AH, Speirs HJ, Goy C, Adams DJ, Markus MA, Morris BJ (2006). XE7: a novel splicing factor that interacts with ASF/SF2 and ZNF265. Nucleic Acids Res.

[86] Matsukage A, Hirose F, Yoo MA, Yamaguchi M (2008). The DRE/DREF transcriptional regulatory system: a master key for cell proliferation. Biochim Biophys Acta.

[87] Ohshima N, Takahashi M, Hirose F (2003). Identification of a human homologue of the DREF transcription factor with a potential role in regulation of the histone H1 gene. J Biol Chem.

[88] Ellis NA, Tippett P, Petty A, Reid M, Weller PA, Ye TZ, German J, Goodfellow PN, Thomas S, Banting G (1994). PBDX is the XG blood group gene. Nat Genet.

[89] Gelin C, Aubrit F, Phalipon A, Raynal B, Cole S, Kaczorek M, Bernard A (1989). The E2 antigen, a 32 kd glycoprotein involved in T-cell adhesion processes, is the MIC2 gene product. EMBO J.

[90] Fouchet C, Gane P, Cartron JP, Lopez C (2000). Quantitative analysis of XG blood group and CD99 antigens on human red cells. Immunogenetics.

[91] Fouchet C, Gane P, Huet M, Fellous M, Rouger P, Banting G, Cartron JP, Lopez C (2000). A study of the coregulation and tissue specificity of XG and MIC2 gene expression in eukaryotic cells. Blood.

[92] Mulloy JC, Cancelas JA, Filippi MD, Kalfa TA, Guo F, Zheng Y (2010). Rho GTPases in hematopoiesis and hemopathies. Blood.

[93] Gianfrancesco F, Esposito T, Montanini L, Ciccodicola A, Mumm S, Mazzarella R, Rao E, Giglio S, Rappold G, Forabosco A (1998). A novel pseudoautosomal gene encoding a putative GTP-binding protein resides in the vicinity of the Xp/Yp telomere. Hum Mol Genet.

[94] Konishi K, Morishima Y, Ueda E, Kibe Y, Nonomura K, Yamanishi K, Yasuno H (1994). Cataloging of the genes expressed in human keratinocytes: analysis of 607 randomly isolated cDNA sequences. Biochem Biophys Res Commun.

[95] Strausberg RL, Feingold EA, Grouse LH, Derge JG, Klausner RD, Collins FS, Wagner L, Shenmen CM, Schuler GD, Altschul SF, Zeeberg B, Buetow KH, Schaefer CF (2002). Generation and initial analysis of more than 15,000 full-length human and mouse cDNA sequences. Proc Natl Acad Sci U S A.

[96] Yamamoto K, Nagata K, Kida A, Hamaguchi H (2002). Acquired gain of an X chromosome as the sole abnormality in the blast crisis of chronic neutrophilic leukemia. Cancer Genet Cytogenet.

[97] Ozkaynak MF, Ying KL, Laug WE (1988). Triple X chromosome constitution and acute nonlymphocytic leukemia. Cancer Genet Cytogenet.

[98] Moorman AV (2012). The clinical relevance of chromosomal and genomic abnormalities in B-cell precursor acute lymphoblastic leukaemia. Blood Rev.

[99] Paulsson K, Forestier E, Lilljebjorn H, Heldrup J, Behrendtz M, Young BD, Johansson B (2010). Genetic landscape of high hyperdiploid childhood acute lymphoblastic leukemia. Proc Natl Acad Sci U S A.

[100] Forestier E, Izraeli S, Beverloo B, Haas O, Pession A, Michalova K, Stark B, Harrison CJ, Teigler-Schlegel A, Johansson B (2008). Cytogenetic features of acute lymphoblastic and myeloid leukemias in pediatric patients with Down syndrome: an iBFM-SG study. Blood.

[101] Swerdlow AJ, Schoemaker MJ, Higgins CD, Wright AF, Jacobs PA (2005). Cancer incidence and mortality in men with Klinefelter syndrome: a cohort study. J Natl Cancer Inst.

[102] Ganmore I, Smooha G, Izraeli S (2009). Constitutional aneuploidy and cancer predisposition. Hum Mol Genet.

[103] Muhlbacher V, Zenger M, Schnittger S, Weissmann S, Kunze F, Kohlmann A, Bellos F, Kern W, Haferlach T, Haferlach C (2014). Acute lymphoblastic leukemia with low hypodiploid/near triploid karyotype is a specific clinical entity and exhibits a very high TP53 mutation frequency of 93%. Genes Chromosomes Cancer.

[104] Probst FJ, Cooper ML, Cheung SW, Justice MJ (2008). Genotype, phenotype, and karyotype correlation in the XO mouse model of Turner Syndrome. J Hered.

[105] Lane PW, Davisson MT (1990). Patchy fur (Paf), a semidominant X-linked gene associated with a high level of X-Y nondisjunction in male mice. J Hered.

[106] Burgoyne PS, Evans EP (2000). A high frequency of XO offspring from X(Paf)Y* male mice: evidence that the Paf mutation involves an inversion spanning the X PAR boundary. Cytogenet Cell Genet.

[107] Blaschke RJ, Rappold GA (1997). Man to mouse--lessons learned from the distal end of the human X chromosome. Genome Res.

[108] Yang H, Wang H, Jaenisch R (2014). Generating genetically modified mice using CRISPR/Cas-mediated genome engineering. Nat Protoc.

[109] Balaton BP, Brown CJ (2016). Escape Artists of the X Chromosome. Trends Genet.

[110] Heard E, Disteche CM (2006). Dosage compensation in mammals: fine-tuning the expression of the X chromosome. Genes Dev.

[111] Agarwal A, MacKenzie RJ, Pippa R, Eide CA, Oddo J, Tyner JW, Sears R, Vitek MP, Odero MD, Christensen DJ, Druker BJ (2014). Antagonism of SET using OP449 enhances the efficacy of tyrosine kinase inhibitors and overcomes drug resistance in myeloid leukemia. Clin Cancer Res.

